# Heat stress-induced mitochondrial damage and its impact on leukocyte function

**DOI:** 10.1186/s40560-025-00832-9

**Published:** 2025-11-04

**Authors:** Toshiaki Iba, Julie Helms, Isao Nagaoka, Ricard Ferrer, Jerrold H. Levy

**Affiliations:** 1 Faculty of Medical Science, Juntendo University, 6-8-1 Hinode, Urayasu, Chiba 279-0013 Japan; 2 Medicine Faculty, Medical Intensive Care Unit NHC, Strasbourg University, University Hospital Federation (FHU) TARGET, Strasbourg University Hospital, Strasbourg, France; 3 Faculty of Medical Science, Juntendo University, 6-8-1 Hinode, Urayasu, Chiba Japan; 4 Intensive Care Department, Hospital Universitari Vall d’Hebron Universitat Autònoma de Barcelona, Barcelona, Spain; 5 Department of Anesthesiology, Critical Care, and Surgery, Duke University School of Medicine, Durham, NC USA; 6 INSERM (French National Institute of Health and Medical Research, UMR 1260, Regenerative Nanomedicine (RNM), FMTS, Strasbourg, France

**Keywords:** Heat stress, Leukocyte, Mitochondria, Cell death, Organ dysfunction

## Abstract

Heatstroke is characterized by systemic inflammation, immune dysregulation, and multiorgan failure, in which mitochondrial damage in leukocytes plays a pivotal role. This review examines the mechanisms by which heat stress induces leukocyte mitochondrial dysfunction and its downstream effects on immunity, coagulation, and organ integrity. Exposure to heat stress activates leukocytes through damage-associated molecular patterns (DAMPs), triggering the release of proinflammatory cytokines, reactive oxygen species (ROS), and neutrophil extracellular traps (NETs). These responses disrupt endothelial integrity, promote microvascular thrombosis, and contribute to the development of disseminated intravascular coagulation (DIC). Prolonged heat exposure further shifts the immune landscape toward immunosuppression, marked by monocyte deactivation and lymphocyte apoptosis. Mitochondrial dysfunction is central to this biphasic immune response. Heat stress reduces mitochondrial membrane potential, increases ROS production, and promotes the release of mitochondrial DNA and cytochrome c, amplifying inflammation and initiating cell death pathways, including apoptosis, pyroptosis, and ferroptosis. Biomarkers such as reduced mitochondrial membrane potential (ΔΨm), elevated mitochondrial ROS, cytochrome c, circulating mitochondrial DNA (mtDNA), and altered expression of mitophagy regulators (e.g., PINK1 and Parkin) provide insights into mitochondrial integrity and function in leukocytes. In addition to immune disruption, mitochondrial injury exacerbates coagulation abnormalities by promoting platelet activation and endothelial dysfunction, fostering a prothrombotic environment. In the microcirculation, leukocyte adhesion, NET formation, and endothelial damage create a self-amplifying cycle of ischemia and inflammation, ultimately leading to organ dysfunction, including hepatic failure, acute kidney injury, acute lung injury, and gastrointestinal barrier breakdown. Therapeutic strategies aimed at preserving mitochondrial function include antioxidants (e.g., N-acetylcysteine and MitoQ), mitochondrial biogenesis inducers (e.g., PGC-1α activators), and mitophagy enhancers. Understanding the central role of leukocyte mitochondrial damage in heat stress provides a foundation for the development of targeted diagnostics and interventions to prevent organ failure and improve clinical outcomes.

**Supplementary Information:**

The online version contains supplementary material available at 10.1186/s40560-025-00832-9.

## Introduction

Under heat stress, leukocytes undergo profound functional and phenotypic changes that contribute to systemic inflammation and organ dysfunction, forming a critical component of heat stroke pathophysiology [1]. Initially, exposure to excessive heat triggers the release of damage-associated molecular patterns (DAMPs), such as heat shock protein (HSP) 70, HSP72, and High Mobility Group Box 1 (HMGB1), from injured tissues, which in turn activate pattern recognition receptors (PRRs), such as toll-like receptors (TLRs) on leukocytes [2]. This leads to rapid activation of neutrophils and monocytes, resulting in the release of proinflammatory cytokines, including IL-1β, IL-6, and TNF-α. These cytokines amplify the systemic inflammatory response, further promoting endothelial activation, increased vascular permeability, and further leucocyte recruitment into tissues [3].

Neutrophils play a central role by releasing reactive oxygen species (ROS), proteases, and forming neutrophil extracellular traps (NETs), which, while initially protective, can damage endothelial cells and exacerbate microvascular thrombosis [4]. Concomitantly, the heat stress induces lymphocyte apoptosis, particularly of CD4^+^ and CD8^+^ T cells, leading to a compromised adaptive immune response [5]. As the condition progresses, many patients shift from a hyperinflammatory state toward immunoparalysis, characterized by monocyte deactivation and further lymphopenia [6], increasing susceptibility to secondary infections and delaying inflammation resolution.

In addition, altered leukocyte trafficking and dysregulated expression of adhesion molecules further disrupt immune surveillance and tissue repair. The number and function of leukocytes can vary during the clinical course: an early leukocytosis with neutrophilia may be followed by leukopenia, particularly in severe or prolonged cases of heat stress, with these changes often correlating with a worse prognosis [7]. Importantly, many of these leukocyte responses mimic those seen in sepsis and sterile systemic inflammatory response syndrome (SIRS), highlighting the overlap between infectious and non-infectious inflammatory triggers [8]. Moreover, the dysregulated immune response under heat stress contributes to coagulation abnormalities, such as disseminated intravascular coagulation (DIC), further complicating clinical outcomes [9]. Emerging studies also suggest that mitochondrial dysfunction and oxidative stress within leukocytes themselves may impair their phagocytic and signaling capacities, reinforcing both the inflammatory and immunosuppressive phases of heat-related illness [10].

Taken together, leukocyte dysregulation under heat stress reflects a complex immune response in which hyperinflammatory and immunosuppressive features may coexist. While a biphasic model of early hyperactivation followed by subsequent exhaustion has been described, recent evidence, including reduced monocyte HLA–DR expression at admission in exertional heatstroke [6] and similar findings in sepsis, indicates that inflammation and immunosuppression can overlap from the outset. This evolving view underscores the heterogeneity of immune responses in heat stroke and highlights the need for further investigation.

This review provides an updated synthesis of the effect of heat stress on mitochondria and its consequences for leukocyte dysfunction and death.

## Mechanism

### Mitochondrial damage

Heat stress causes profound mitochondrial dysfunction in leukocytes, acting as a central mediator of immune dysregulation and systemic inflammation in heat-related illnesses [11, 12]. Mitochondria are crucial for energy production, maintaining redox balance, and regulating immune signaling. Elevated temperatures disrupt mitochondrial structure and function, compromising their ability to support normal leukocyte activity [13, 14].

One of the primary consequences of heat exposure is the loss of mitochondrial membrane potential (ΔΨm), which impairs ATP synthesis and diminishes energy-dependent leukocyte processes, such as migration, phagocytosis, and cytokine production [11, 13]. Heat stress also accelerates mitochondrial reactive oxygen species (mtROS) generation, resulting in oxidative damage that affects mitochondrial membranes, enzymes, and DNA [13, 15]. Although mtROS are involved in antimicrobial responses under physiological conditions, their excessive accumulation promotes cellular injury and proinflammatory signaling [16, 17].

**Fig. 1:**
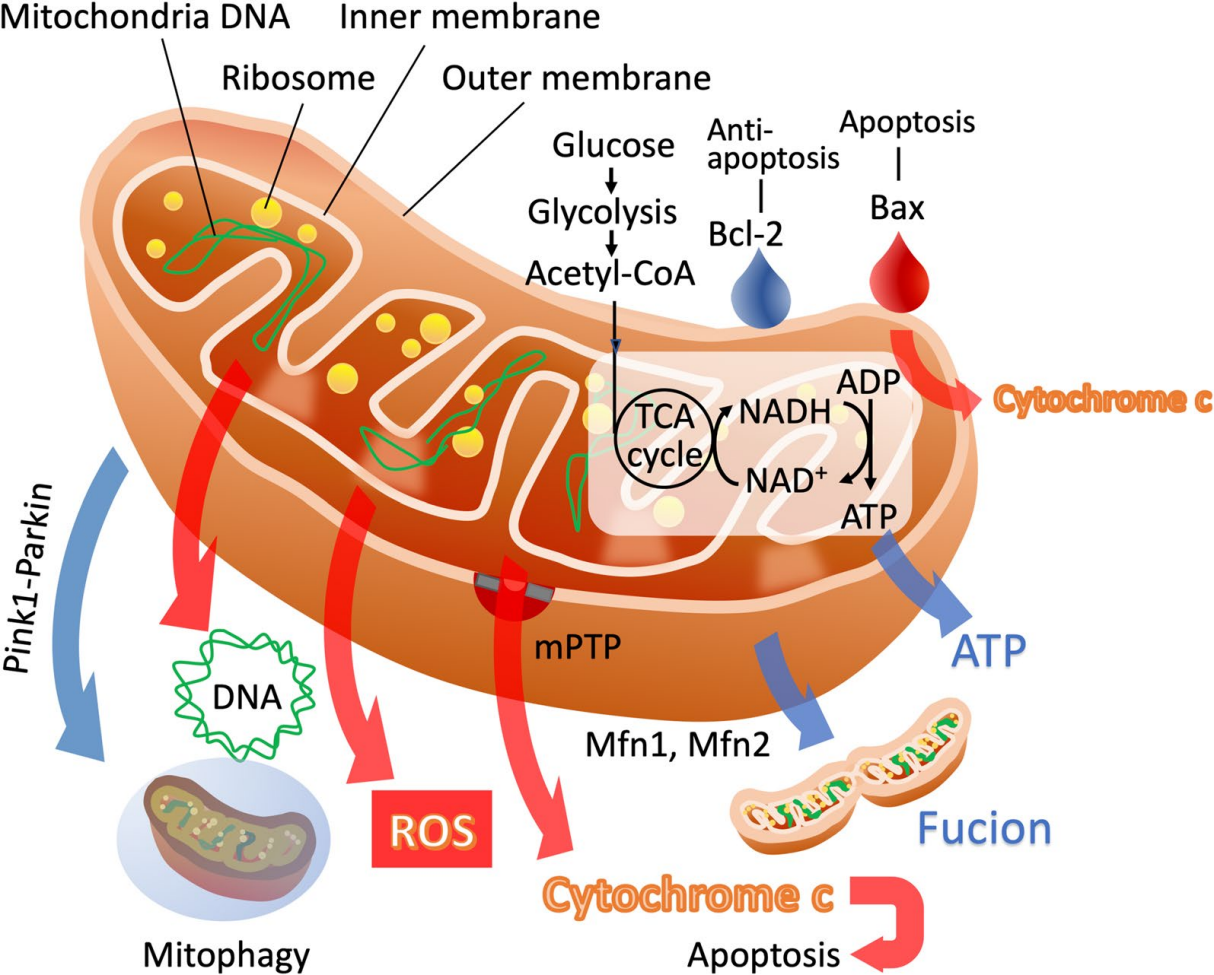
Mitochondrial responses to heat stress. Heat stress induces mitochondrial damage, leading to multiple intracellular responses. The figure illustrates the mitochondrion's inner and outer membranes, mitochondrial DNA (mtDNA), ribosomes, and critical biomolecules, such as cytochrome c, adenosine triphosphate (ATP), and reactive oxygen species (ROS). Heat-induced mitochondrial dysfunction triggers the release of ROS and cytochrome c, leading to oxidative damage, mitochondrial DNA disruption, and apoptosis. The Tricarboxylic Acid (TCA) cycle is impaired, reducing ATP production. Damaged mitochondria are targeted for mitophagy, primarily through the Pink1–Parkin pathway. Meanwhile, the dynamic processes of mitochondrial fusion and fission modulate the mitochondrial network to maintain homeostasis or propagate damage under excessive stress. Bcl-2: B-cell lymphoma 2; Bax: Bcl-2-associated X protein; mPTP: mitochondrial permeability transition pore; Mfn: mitochondria fusion; Blue arrow: protective response; Red arrow: injurious response

Damaged mitochondria release immunostimulatory molecules, such as mitochondrial DNA (mtDNA) and cytochrome c, which act as DAMPs (Fig. 1). These molecules activate innate immune receptors, such as TLR9 and cGAS–STING (cyclic GMP–AMP synthase–stimulator of interferon genes), amplifying inflammation and triggering cell death pathways, including apoptosis and pyroptosis [18, 19]. For instance, mitochondrial outer membrane permeabilization leads to the release of cytochrome c and the initiation of the intrinsic apoptotic cascade, reducing lymphocyte viability and contributing to immunosuppression [20, 21].

The impact of mitochondrial damage varies across leukocyte subsets. Neutrophils exhibit impaired chemotaxis and altered NET formation under mitochondrial stress, while monocytes demonstrate reduced antigen-presenting capacity and disrupted metabolic reprogramming [22]. These changes compromise immune surveillance and resolution of inflammation, contributing to the biphasic immune response seen in heatstroke: an initial hyperinflammatory phase followed by immunosuppression [6, 23]. On the other hand, recent evidence suggests mitochondrial dysfunction in heatstroke operates within a bidirectional loop rather than a single linear cascade. Direct thermal stress can impair electron transport chain function, promote, and precipitate permeability transition, leading to loss of membrane potential and excess ROS generation [11].

**Fig. 2:**
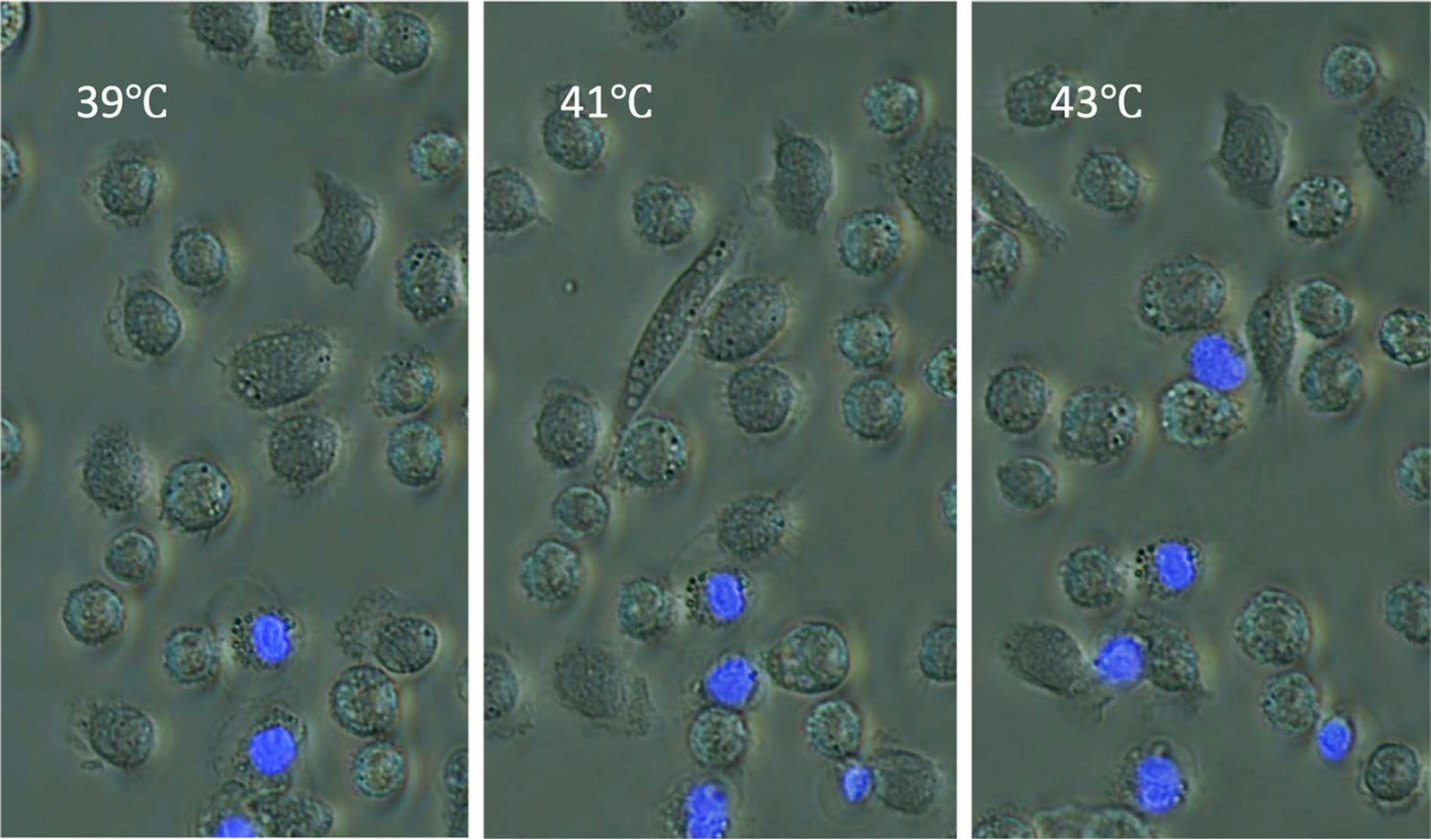
Temperature-dependent increase in leukocyte cell death following heat exposure. Leukocytes were isolated from the abdominal cavity and cultured. The temperature of the culture medium was increased almost linearly from 37 ℃ to 43 ℃ in 3 h (The temperatures 39 ℃, 41 ℃, and 43 ℃ mean times 1, 2, and 3 h) at temperatures ranging from 39 °C to 43 °C. Intracellular DNA was stained with DAPI (4′,6-diamidino-2-phenylindole, blue), and phase-contrast imaging was used to assess cellular morphology. While phase-contrast images revealed minimal morphological changes even at 43 °C, DAPI staining showed a progressive increase in the number of positively stained cells with rising temperature, indicating a temperature-dependent increase in cell death

**Fig. 3:**
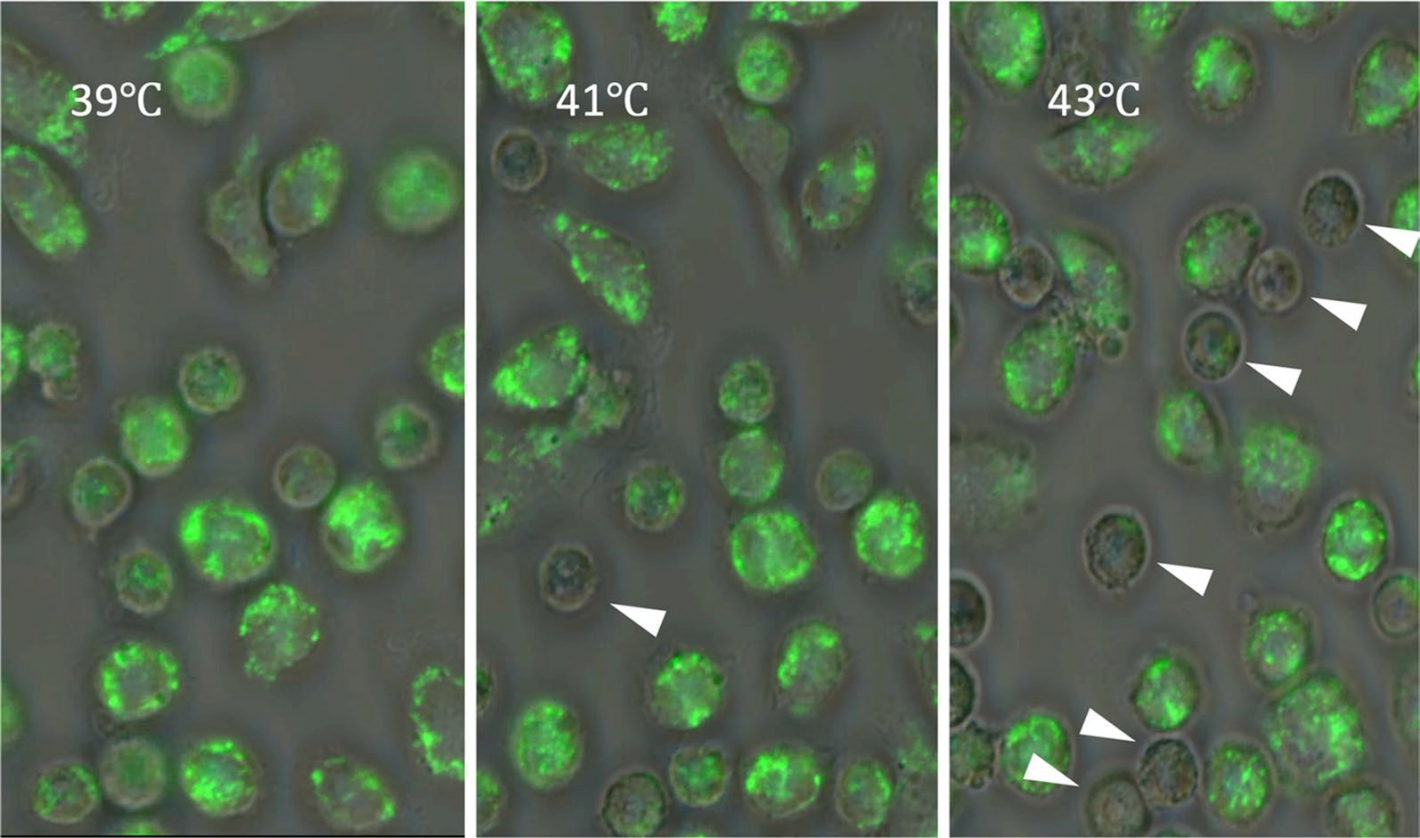
Temperature-dependent loss of mitochondrial staining in leukocytes. Mitochondria in leukocytes were visualized using MitoBright LT^™^ (Dojindo, Kumamoto, Japan), a small-molecule fluorescent dye that selectively stains mitochondria via electrostatic interactions. This staining method allows for the assessment of mitochondrial distribution, abundance, and morphology. Leukocytes were exposed to increasing temperatures (from 39 °C to 43 °C in 3 h), and mitochondrial integrity was assessed by fluorescence microscopy. As the temperature increased, a growing number of cells showed a loss of mitochondrial staining (indicated by arrows), suggesting heat-induced mitochondrial depolarization or degradation

**Fig. 4:**
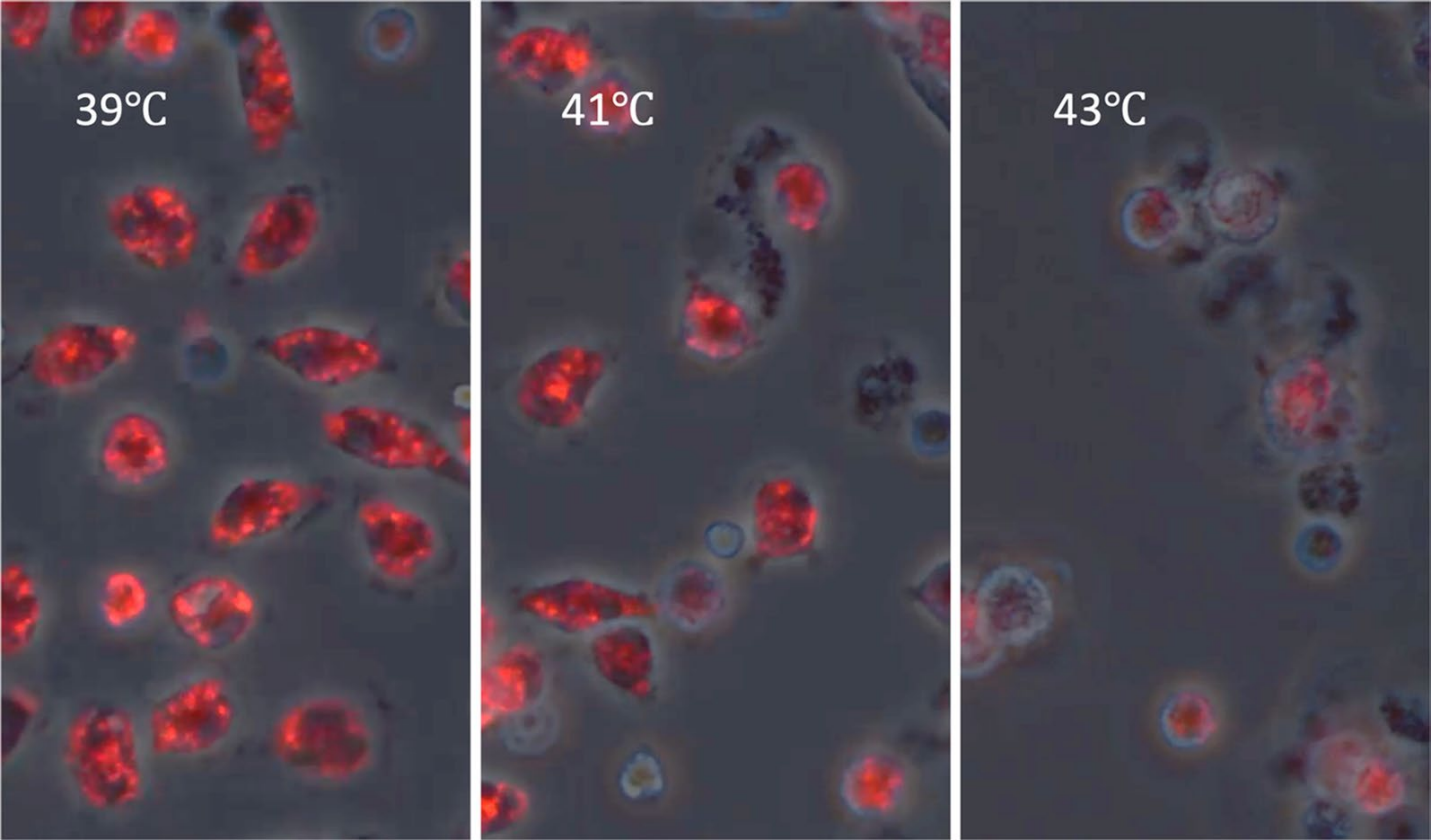
Heat stress induces a temperature-dependent loss of mitochondrial membrane potential. Mitochondria are critical for cellular energy production through the generation of adenosine triphosphate (ATP). Their function is tightly linked to mitochondrial membrane potential (Δψm), which reflects the organelle’s capacity to produce ATP. JC-1 (5,5′,6,6′-tetrachloro-1,1′,3,3′-tetraethylbenzimidazolylcarbocyanine iodide), a cationic fluorescent dye, was used to visualize Δψm. Under normal conditions, JC-1 aggregates in mitochondria and emits red fluorescence, whereas depolarized mitochondria show green fluorescence due to monomeric JC-1. The figure illustrates a progressive, temperature-dependent decline in membrane potential with increasing heat stress (from 39 °C to 43 °C in 3 h), suggesting mitochondrial dysfunction under elevated thermal conditions. Loss of mitochondrial membrane potential precedes mitochondrial degradation and cell death, suggesting it may play a causative role in initiating these processes

Compounding this injury, heat stress impairs mitophagy, the selective degradation of dysfunctional mitochondria, allowing damaged organelles to accumulate [12, 24]. This further promotes oxidative stress, cytokine production, and leukocyte dysfunction. Experimental studies using microscopy and functional assays confirm the presence of mitochondrial fragmentation, swelling, and depolarization in leukocytes exposed to heat [25]. The longitudinal experimental observations provide sequential evidence that decreased mitochondrial depolarization precedes mitochondrial degradation and subsequent leukocyte death, thereby supporting the temporal cascade (Figs. 2, 3 and 4, Suppl. Figures 1, 2 and 3).

Altogether, these mitochondrial alterations in leukocytes under heat stress initiate a feedback loop of energy depletion, inflammation, and immune cell death, contributing to multiorgan dysfunction and poor clinical outcomes [14, 26]. Targeting mitochondrial integrity and function may offer novel therapeutic avenues for managing heat-related syndromes [27, 28].

### Leukocyte cell death under heat stress

Leukocyte death is a pivotal consequence of heat stress and plays a central role in immune dysfunction, systemic inflammation, and organ failure. Various modes of cell death—apoptosis, pyroptosis, necrosis, necroptosis, ferroptosis, and NETosis—can be activated in leukocytes, depending on the intensity of stress, mitochondrial status, and metabolic context [29, 30]. Among these, mitochondria-mediated apoptosis is a major contributor, especially in lymphocytes. Heat stress disrupts mitochondrial membrane integrity, promoting the release of cytochrome c and the activation of the intrinsic apoptotic cascade through caspase-9 and caspase-3, which leads to DNA fragmentation and cellular dismantling [20, 21, 31]. This reduces lymphocyte populations, notably CD4^+^ and CD8^+^ T cells, impairing adaptive immunity and predisposing patients to secondary infections [32, 33].

Beyond apoptosis, mitochondrial dysfunction also predisposes monocytes and neutrophils to necrotic and necroptotic death, particularly when ATP production is compromised [34, 35]. These forms of cell death release intracellular DAMPs, including mtDNA, which propagates inflammation via TLR9 and cGAS–STING pathways, further amplifying systemic immune activation [18, 19].

Pyroptosis, driven by inflammasome activation (e.g., NLRP3), is especially relevant in monocytes and macrophages under thermal injury [36]. Heat-induced mitochondrial ROS promotes inflammasome assembly, activating gasdermin D and inducing pore formation, resulting in IL-1β and IL-18 release [16, 37]. This proinflammatory cell death not only exacerbates leukocyte dysfunction but also reinforces endothelial damage and vascular leakage, linking directly to the coagulopathy described in Sect. “Coagulation disorders and mitochondrial damage under heat stress”.

NETosis, a neutrophil-specific response to oxidative stress, involves the release of chromatin-based NETs. Although NETs can sequester pathogens, their excessive release under heat stress damages endothelial barriers and promotes thrombosis, contributing to DIC [4, 38]. These processes highlight a convergence between cell death and coagulation, emphasizing the need for integrated therapeutic approaches.

Furthermore, ferroptosis, an iron-dependent, lipid peroxidation-driven cell death, has been observed under oxidative thermal stress, although its role in leukocytes remains under investigation [29]. The balance between survival and cell death is modulated by mitochondrial integrity, redox status, and anti-apoptotic factors, such as Bcl-2 and HSP70, whose expression can delay or inhibit death signaling [39].

In clinical settings, elevated leukocyte apoptosis and impaired monocyte function correlate with poor outcomes in heatstroke patients [35]. As such, leukocyte cell death is not merely a marker of heat-related damage but also a driver of disease progression. Therapeutic interventions aimed at preserving mitochondrial function, inhibiting inflammasome activation, or blocking executioner caspases may hold promise in attenuating immune collapse and mitigating organ injury in severe heat-related illnesses.

### Coagulation disorders and mitochondrial damage under heat stress

Coagulation abnormalities are a critical complication of heat stress and are closely linked to mitochondrial dysfunction across various cell types, including leukocytes, endothelial cells, and platelets. Mitochondria not only regulate cellular energy metabolism but also modulate redox signaling, inflammation, and programmed cell death, all of which influence hemostasis. Under heat stress, mitochondrial injury disrupts ΔΨm, impairs oxidative phosphorylation, and accelerates the generation of mtROS, thereby promoting procoagulant responses [11, 17, 27, 35].

In endothelial cells, mitochondrial damage increases oxidative stress, reduces ATP production, and impairs endothelial barrier function. This leads to enhanced vascular permeability and the exposure of subendothelial procoagulant factors, such as tissue factor (TF) and collagen, which initiate the extrinsic and intrinsic coagulation cascades [15, 27]. Concurrently, mitochondrial dysfunction promotes the release of circulating mtDNA, a potent DAMP that activates innate immune receptors, such as TLR9 and cGAS–STING, upregulating TF expression and priming monocytes and platelets for procoagulant activity [18, 40].

Platelets, when exposed to elevated temperatures, undergo mitochondrial depolarization, mtROS accumulation, and apoptosis-like changes. These mitochondrial alterations enhance platelet activation, aggregation, and the release of procoagulant microvesicles, collectively increasing thrombin generation and fibrin deposition [35, 41]. In parallel, leukocyte activation under heat stress contributes to NET formation, which further promotes microvascular thrombosis and the development of DIC [4, 38].

Inflammatory cytokines (e.g., IL-1β and IL-18) produced by pyroptotic monocytes and other immune cells exacerbate endothelial activation and coagulation, establishing a feed-forward loop between inflammation and thrombosis [37, 42]. This process is further aggravated by suppressed anticoagulant systems, including reduced expression or activity of antithrombin, protein C, and tissue factor pathway inhibitor (TFPI), all of which are negatively influenced by oxidative stress and mitochondrial dysfunction [5, 43].

At the same time, heat stress impairs endothelial production of anticoagulant and fibrinolytic mediators, such as thrombomodulin and prostacyclin, due to mitochondrial ATP depletion and ROS-induced signaling defects [5, 15]. These imbalances tip the hemostatic scale toward a prothrombotic state. In advanced stages, this may evolve into consumptive coagulopathy, where platelets and coagulation factors are depleted, increasing the risk of bleeding—an ominous feature in fulminant heatstroke [7].

Taken together, mitochondrial dysfunction under heat stress serves as a central link connecting oxidative stress, inflammatory signaling, and coagulation activation. The interaction between mitochondrial injury and coagulopathy creates a vicious cycle in which inflammation and microthrombosis impair tissue perfusion, exacerbate organ damage, and further perpetuate mitochondrial dysfunction [12, 26]. Targeted therapies aimed at preserving mitochondrial integrity—such as antioxidants, mitochondrial reactive oxygen species scavengers, and mitochondrial membrane stabilizers—may offer promising strategies to interrupt this cycle and reduce coagulation-related complications in severe heat-related illnesses. Biomarkers such as circulating mtDNA, platelet activation markers, and thrombin–antithrombin complexes may aid in the early detection and monitoring of heat-induced coagulopathy [40, 41].

### Microcirculatory damage under heat stress

**Fig. 5:**
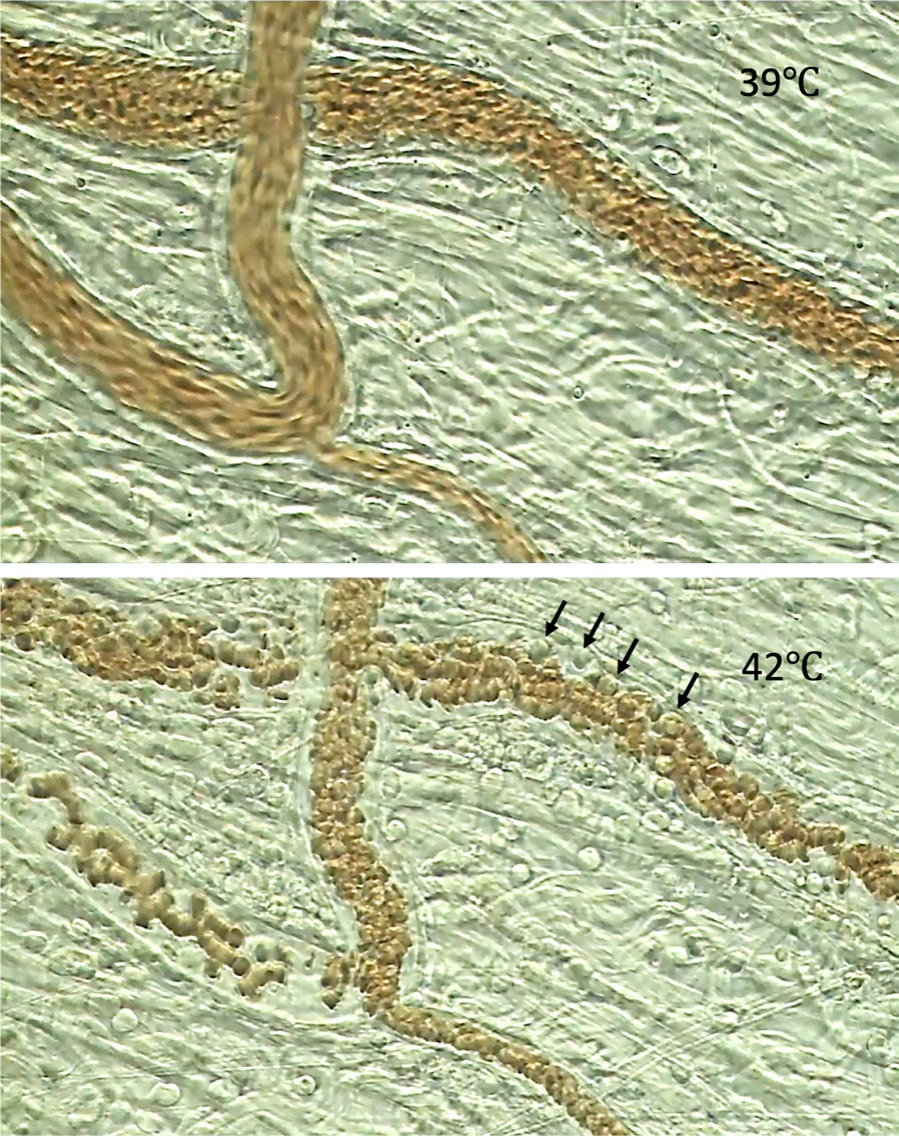
Temperature-dependent alterations in mesenteric microcirculation during heat stress. Intravital microscopy was used to assess mesenteric microcirculation in a rat subjected to progressive heat exposure. At a core body temperature of 39 °C (top panel), blood flow appeared smooth with minimal leukocyte–endothelium interaction. As the temperature increased, leukocytes (indicated by black arrows) became rounded and began adhering to the vascular endothelium. At 42 °C (bottom panel), significant leukocyte adhesion led to disrupted blood flow, reflecting microcirculatory disturbance induced by heat stress

Microcirculatory dysfunction is a fundamental pathophysiological feature of heat-related illness, linking leukocyte activation, endothelial injury, and organ failure. The microcirculation, including capillaries, arterioles, and venules, plays a critical role in oxygen and nutrient delivery and waste removal. Under heat stress, this finely tuned network is disrupted by thermal injury, hemodynamic instability, and systemic inflammation, leading to tissue hypoperfusion and metabolic collapse [42, 44] (Fig. 5), (Suppl. Figure 4).

Direct thermal damage to endothelial cells impairs cytoskeletal organization, induces protein denaturation, and disrupts intercellular junctions, resulting in barrier breakdown and increased vascular permeability [45]. These changes are compounded by oxidative stress and ATP depletion caused by mitochondrial dysfunction within endothelial cells, which further compromise the integrity of tight junctions and increase capillary leakage [12, 27]. Simultaneously, activated leukocytes, especially neutrophils and monocytes, adhere to the damaged endothelium via upregulated adhesion molecules, such as ICAM-1 and VCAM-1, triggering localized inflammation and amplifying vascular injury [12, 46].

A hallmark of microcirculatory damage under heat stress is the formation of NETs. While NETs help trap pathogens, their excessive release obstructs capillary flow, promotes microthrombosis, and directly injures endothelial cells, contributing to DIC and further exacerbating vascular dysfunction [4, 47, 48].

These events culminate in capillary leak syndrome, leading to tissue edema, impaired oxygen diffusion, and localized hypoxia. Organs with high metabolic demands, such as the brain, liver, kidneys, and lungs, are particularly vulnerable to these effects, which manifest clinically as encephalopathy, hepatic injury, acute kidney injury, and non-cardiogenic pulmonary edema [26, 49].

Hemodynamic instability, resulting from systemic vasodilation and hypovolemia, further impairs microvascular perfusion. Even after normalization of systemic blood pressure, heterogeneous perfusion and “no-reflow” phenomena persist due to endothelial swelling, microthrombi formation, and leukocyte plugging—especially in renal and splanchnic territories [50, 51]. These regional ischemic zones contribute to irreversible organ damage and are not corrected by conventional fluid resuscitation or vasopressor therapy alone.

Mitochondrial damage in both leukocytes and endothelial cells plays a central role in this process by driving oxidative injury, reducing cellular resilience, and fueling proinflammatory and procoagulant signaling [12, 42]. The interplay between mitochondrial dysfunction, leukocyte activation, and endothelial disruption forms a self-perpetuating cycle of microcirculatory collapse.

In summary, microcirculatory injury under heat stress is a multifactorial process involving thermal endothelial injury, mitochondrial failure, immune cell activation, and microthrombosis. These alterations lead to inadequate tissue perfusion, organ ischemia, and ultimately multiorgan failure. Therapeutic strategies aimed at preserving endothelial and mitochondrial function, limiting leukocyte activation, and restoring capillary integrity are crucial for mitigating these downstream effects and improving outcomes in severe heat-related illnesses.

### Organ dysfunction under heat stress and its relation to leukocyte damage

Multiorgan dysfunction is a defining feature of severe heat-related illness and is closely linked to leukocyte activation, mitochondrial injury, and systemic inflammation. Upon exposure to extreme temperatures, injured tissues release DAMPs that trigger leukocyte activation via pattern recognition receptors, initiating a cascade of proinflammatory responses. Activated leukocytes, particularly neutrophils and monocytes, release cytokines (e.g., IL-1β and TNF-α), ROS, and proteolytic enzymes, contributing to endothelial injury, capillary leak, and microvascular thrombosis [1, 4, 52].

In parallel, mitochondrial dysfunction within leukocytes compromises ATP production and redox balance, promoting cell death through apoptosis, pyroptosis, and NETosis [12, 19, 20]. These forms of leukocyte death not only reduce immune cell numbers, especially lymphocytes, but also release secondary DAMPs and inflammatory mediators, amplifying systemic inflammation and propagating tissue injury. This feedback loop between mitochondrial damage and immune dysregulation accelerates organ failure across multiple systems.

The kidneys are especially vulnerable, frequently exhibiting acute tubular necrosis and ischemia due to both hypoperfusion and leukocyte-driven inflammation within the renal microvasculature [26]. In the liver, histological changes include sinusoidal congestion, leukocyte infiltration, and hepatocellular necrosis, reflecting both systemic inflammation and oxidative damage [53]. Pulmonary complications, including non-cardiogenic pulmonary edema and acute respiratory distress syndrome (ARDS), result from increased endothelial permeability and leukocyte-induced alveolar–capillary barrier disruption [54]. In the central nervous system, elevated cytokine levels and compromised blood–brain barrier integrity contribute to neuronal dysfunction and cerebral edema, manifesting clinically as confusion, delirium, or coma [55].

In addition, heat stress impairs gastrointestinal barrier function, allowing bacterial translocation and further activation of innate immune responses, which can exacerbate systemic inflammation and worsen organ injury [56]. The cumulative impact of leukocyte activation, endothelial dysfunction, and mitochondrial impairment results in widespread tissue hypoxia, metabolic failure, and ultimately, multiorgan failure.

A distinctive feature of the immune response to heat stress is its biphasic nature. The initial hyperinflammatory phase is characterized by massive cytokine release and leukocyte activation, while the later immunosuppressive phase involves lymphocyte apoptosis and monocyte deactivation (e.g., reduced HLA–DR expression), resulting in impaired pathogen clearance and increased susceptibility to secondary infections [6, 23]. Mitochondrial injury plays a crucial role in both phases, promoting early immune activation through the release of DAMPs and driving later immunoparalysis via energy failure and the apoptotic loss of immune cells.

## Biomarkers for mitochondrial damage

Identifying biomarkers of mitochondrial dysfunction in leukocytes provides valuable insight into the pathophysiology of heat-related illnesses and offers tools for diagnosis and therapeutic monitoring [57]. Mitochondrial damage affects energy production, redox homeostasis, and immune signaling, and several measurable indicators have been developed to assess these alterations.

One major class of biomarkers involves ΔΨm, a surrogate for mitochondrial function and ATP-generating capacity. Decreased ΔΨm can be detected using fluorescent dyes, such as JC-1, TMRE (tetramethylrhodamine ethyl ester), or Rhodamine 123 via flow cytometry, indicating compromised mitochondrial integrity [58, 59]. A decline in ΔΨm is correlated with impaired leukocyte function and increased susceptibility to programmed cell death [13, 21].

MtROS levels, another key indicator of oxidative stress, are commonly measured using probes, such as MitoSOX Red^™^ (Mitochondrial Superoxide Indicator, for live-cell imaging, ThermoFisher Scientific Inc., Waltham, MA) [53]. Elevated mtROS levels reflect excessive oxidative activity and are associated with leukocyte dysfunction, inflammasome activation, and inflammatory cell death [16, 17]. However, the clinical application of mtROS as a biomarker is limited by its extremely short half-life, and thus surrogate indicators of oxidative stress may provide more reliable diagnostic value.

Similarly, the release of cytochrome c into the cytosol or plasma serves as a biomarker for mitochondrial outer membrane permeabilization and the initiation of intrinsic apoptosis [60].

Circulating or intracellular mtDNA is a potent DAMP and can be quantified via qPCR techniques. Elevated mtDNA levels are indicative of mitochondrial disruption and systemic inflammation [40, 61]. These changes may also point to impaired mitophagy and the accumulation of damaged mitochondria [12]. Complementary markers include mitophagy-related proteins, such as PINK1 (PTEN-induced kinase 1), Parkin, and LC3B, which can be measured via immunoblotting or flow cytometry. The altered expression of these proteins reflects the status of mitochondrial quality control and correlates with disease severity [24, 62].

In addition to structural and redox biomarkers, metabolic indicators such as reduced ATP content or altered NAD^+^/NADH ratios provide insight into mitochondrial metabolic failure. These can be evaluated using enzymatic or luminescent assays, offering insight into the energetic capacity of leukocytes during heat stress [63, 64].

Collectively, these biomarkers offer a comprehensive assessment of mitochondrial health, encompassing functional, structural, and metabolic aspects. Their integration into clinical and research settings may aid in early diagnosis, risk stratification, and evaluation of therapeutic responses targeting mitochondrial preservation in heat-related illnesses [6, 33].

Although ΔΨm, mtROS, and circulating mtDNA have been proposed as promising indicators of mitochondrial dysfunction, their translation into routine clinical use faces several obstacles. Measurement of ΔΨm typically requires fluorescent probes in freshly isolated cells, limiting its applicability at the bedside, where real time, minimally invasive assays are preferable. Similarly, direct quantification of mtROS is hampered by their extremely short half-life and the need for specialized probes or imaging platforms, restricting current use to research laboratories. Circulating cell-free mtDNA can be measured using quantitative PCR or next-generation sequencing approaches, but results are influenced by pre-analytical variables (sample type, processing time, storage) and analytical variability across laboratories [5]. Standardization of protocols and establishment of clinically meaningful cutoffs remain unresolved. Cost is another consideration, as high-throughput molecular assays may not be feasible in all healthcare systems.

## Management of mitochondrial damage under heat stress

Effective management of mitochondrial damage is crucial for improving outcomes in heat-related illnesses, where mitochondrial dysfunction leads to immune dysregulation, coagulopathy, and organ failure. Heat stress disrupts ΔΨm, enhances the production of mtROS, and induces the release of pro-apoptotic and proinflammatory molecules, such as cytochrome c and mtDNA, initiating cascades of inflammation, cell death, and tissue injury [11, 12, 65].

Therapeutic strategies aim to preserve or restore mitochondrial integrity, limit oxidative damage, and promote mitochondrial quality control mechanisms. One core approach involves antioxidant therapy, which targets excessive mtROS and prevents oxidative injury to mitochondrial membranes, proteins, and DNA. Agents such as N-acetylcysteine (NAC), coenzyme Q10, vitamin C, and mitochondria-targeted antioxidants like mitoquinone (MitoQ) have shown efficacy in experimental models, reducing oxidative stress and preserving mitochondrial function under thermal injury [66–68] (Table 1).

**Table 1:** Mitochondria-targeted therapies

Agent (class)	Mechanism (target)	Key experimental/clinical evidence	Clinical status (2025)	Main limitations
MitoQ/Mitoquinone	Mitochondria-targeted antioxidant; scavenges ROS	Preclinical organ protection; small human feasibility studies show biomarker modulation	Investigational; small clinical trials ongoing, outcome benefit unproven	Clinical efficacy not established; PK in critically ill uncertain; tissue delivery may be limited
SS-31/Elamipretide	Cardiolipin-binding peptide; stabilizes ETC, reduces ROS, preserves ATP	Robust preclinical cardioprotection; phase 2–3 trials in myopathies and HF show mixed signals	Advanced clinical development in rare mitochondrial disorders, cardiomyopathies	High cost; neutral results in some trials; dosing in shock unvalidated
Resveratrol/PGC-1α activators	SIRT1 activator; enhances PGC-1α → mitochondrial biogenesis	Multiple preclinical studies; small RCTs with heterogeneous biomarker effects	Supplement available; clinical trials heterogeneous; not an approved therapy	Poor bioavailability; slow mechanism may limit use in acute illness
AICAR (AMPK activator)	Activates AMPK; stimulates mitophagy, biogenesis, metabolic reprogramming	Preclinical protection against mitochondrial dysfunction and stress; limited translational work	Experimental; occasional early human pharmacology studies	Limited human safety/PK data; optimal dosing in critical illness unknown
Melatonin	Direct antioxidant; regulates mitophagy, fusion/fission, mitochondrial enzymes	Extensive preclinical; human trials in oxidative stress show biomarker/physiologic effects	Widely available, safe, inexpensive; multiple small clinical trials	Variable dosing; limited data on mortality or organ protection in shock
N-acetylcysteine (NAC)	Replenishes glutathione, scavenges ROS, modulates redox	Long history of clinical use; RCTs in sepsis/SIRS show mixed outcomes	Approved for acetaminophen toxicity; widely available	No consensus on dose for mitochondrial rescue; inconsistent outcome benefit
Cyclosporine A	Inhibits cyclophilin D → prevents mPTP opening	Strong preclinical ischemia/reperfusion protection; clinical trials in MI largely neutral	Approved immunosuppressant; tested experimentally in acute injury	Immunosuppressive, nephrotoxic; neutral outcomes in cardioprotection trials

ROS: reactive oxygen species, PK: pharmacokinetics, HF: heart failure, SIRT1: silent information regulator 1, PGC-1α: peroxisome proliferator-activated receptor gamma coactivator 1-alpha, RCTs: randomized controlled trials, AICAR: 5-Aminoimidazole-4-carboxamide ribotide activator, AMPK: adenosine monophosphate-activated protein kinase, NAC: N-acetylcysteine, SIRS: systemic inflammatory response syndrome, mPTP: mitochondrial permeability transition pore, MI: myocardial infarction

Enhancing mitochondrial biogenesis is another key therapeutic avenue. Pharmacologic activators of PGC-1α (peroxisome proliferator-activated receptor gamma coactivator 1-alpha), such as resveratrol, AICAR (5-Aminoimidazole-4-carboxamide ribotide), and melatonin, stimulate mitochondrial replication and metabolic recovery, improving cellular energy balance during and after heat stress [69–71]. Similarly, supporting mitophagy, the selective clearance of damaged mitochondria, is crucial for maintaining mitochondrial homeostasis and preventing inflammatory signaling. Compounds that activate the PINK1/Parkin pathway or AMPK (AMP-activated protein kinase) signaling may help eliminate dysfunctional mitochondria and reduce leukocyte-mediated tissue injury [24].

Mitochondria-targeted peptides, such as SS-31 (elamipretide), represent an emerging class of therapies. SS-31 localizes to the inner mitochondrial membrane, where it stabilizes cardiolipin, reduces membrane permeability, and preserves ATP production under stress conditions [72]. In addition, inhibitors of the mitochondrial permeability transition pore (mPTP), such as cyclosporin A, may prevent mitochondrial swelling, cytochrome c release, and necrotic cell death in heat-stressed tissues [73].

Beyond direct mitochondrial interventions, supportive care remains essential. Rapid cooling is the most effective method to prevent further mitochondrial damage by halting the thermal insult. Maintenance of adequate tissue perfusion through fluid resuscitation, vasopressors, and, if needed, renal replacement therapy is critical to minimize secondary ischemic injury. Metabolic support, including glucose, glutamine, or pyruvate, may serve as alternative energy substrates to sustain mitochondrial function during recovery [64].

Finally, biomarkers of mitochondrial dysfunction, such as circulating mtDNA, reduced ΔΨm in leukocytes, or elevated mtROS, may aid in early diagnosis, risk stratification, and therapeutic monitoring in clinical settings [6, 61]. Incorporating these markers into clinical workflows could enable precision-targeted interventions and improve prognosis in patients with heat-related syndromes.

In summary, managing mitochondrial damage under heat stress requires a multifaceted approach combining antioxidant therapy, mitochondrial biogenesis and mitophagy support, temperature control, and organ support strategies. Targeted mitochondrial therapeutics represent a promising frontier in improving cellular resilience and limiting immune-mediated tissue injury in heat-related illnesses.

## Limitations

The difficulty in researching heatstroke is that it requires extensive use of animal experiments. Although rodent and cell culture studies have provided crucial mechanistic insights into mitochondrial dysfunction, reactive oxygen species generation, and cellular injury during heat stress, their direct translation to human pathophysiology remains challenging. Animal models often employ extreme hyperthermic conditions and genetically homogenous populations that do not fully capture the heterogeneity of human responses. Moreover, the immunological and metabolic responses in rodents may differ substantially from those in humans, limiting the extrapolation of therapeutic targets. Cell culture systems, while valuable for dissecting molecular pathways, lack the complex interactions among immune, vascular, and endocrine systems that shape human disease outcomes. Existing clinical data remain sparse, but reports of mitochondrial damage markers, such as circulating cell-free mitochondrial DNA, in critically ill or heatstroke patients suggest that mitochondrial dysfunction is also relevant in human pathology [5, 74]. Nevertheless, robust human studies, ideally integrating clinical biomarkers with mechanistic endpoints, are urgently needed. Bridging this gap will require translational research that validates preclinical findings in carefully phenotyped patient populations, using advanced molecular and imaging tools.

## Conclusion

Mitochondrial damage in leukocytes plays a central and multifaceted role in the pathogenesis of heat-related illness, linking immune dysfunction, systemic inflammation, microcirculatory collapse, and coagulation abnormalities. Heat stress disrupts mitochondrial homeostasis, leading to energy failure, excessive ROS production, and the release of proinflammatory and prothrombotic mediators. These changes trigger a biphasic immune response—initial hyperactivation followed by immunosuppression—which contributes to multiorgan dysfunction and poor clinical outcomes. Identifying mitochondrial biomarkers such as mtROS, ΔΨm, and mtDNA can offer valuable tools for early diagnosis and monitoring. Therapeutic strategies aimed at preserving mitochondrial integrity, enhancing mitophagy, and limiting oxidative stress hold promise in mitigating disease severity. Interventions such as antioxidant therapy, mitochondrial-targeted agents, and immune modulation may improve cellular resilience and patient recovery. A deeper understanding of mitochondrial–immune interactions under heat stress could pave the way for precision medicine approaches in managing severe heat-related syndromes.

## Supplementary Information

Supplementary Material 1: Changes in leukocyte cell death. Time-lapse observation of leukocytes at 37℃ for 4 hours. Intracellular DNA was stained with DAPI (4‘,6-diamidino-2-phenylindole, blue), and phase-contrast imaging was used to assess cellular morphology. Both phase-contrast view and DAPI staining revealed minimal morphological changes for this period.

Supplementary Material 2: Changes in mitochondrial staining in leukocytes. Mitochondria in leukocytes were visualized using MitoBright LT™ (Dojindo, Kumamoto, Japan) and observed for 4 hours at 37 ℃. This staining method allows for the assessment of mitochondrial distribution and function. As time went on, some cells showed a loss of mitochondrial staining (indicated by arrows).

Supplementary Material 3: Changes in mitochondrial membrane potential. Morphological changes of the leukocytes were observed under the microscope at 37℃ for 4 hours. No significant change was recognized (upper low). JC-1 (5,5',6,6'-tetrachloro-1,1',3,3'-tetraethylbenzimidazolylcarbocyanine iodide), a cationic fluorescent dye, was used to visualize membrane potential (Δψm). Under normal conditions, mitochondrial membrane potential was maintained for 4 hours (lower column).

Supplementary Material 4: Alterations in mesenteric microcirculation. Intravital microscopic observation revealed minimal changes in mesenteric flow in a rat. Blood flow appeared smooth with minimal leukocyte–endothelium interaction throughout the study period.

## Data Availability

Not applicable.

## Abbreviations

DAMPs: Damage-associated molecular patterns
ROS: Reactive oxygen species
NETs: Neutrophil extracellular traps
DIC: Disseminated intravascular coagulation
ΔΨm: Mitochondrial membrane potential
HSP: Heat shock protein
HMGB1: High Mobility Group Box 1
PRRs: Pattern recognition receptors
TLRs: Toll-like receptors
ROS: Reactive oxygen species
NETs: Neutrophil extracellular traps
SIRS: Systemic inflammatory response syndrome
mtROS: Mitochondrial reactive oxygen species
mtDNA: Mitochondrial DNA
cGAS–STING: Cyclic GMP–AMP synthase–stimulator of interferon genes
TF: Tissue factor
TFPI: Tissue factor pathway inhibitor
ARDS: Acute respiratory distress syndrome
TMRE: Tetramethylrhodamine ethyl ester
PINK1: PTEN-induced kinase 1
NAC: N-acetylcysteine
PGC-1α: Peroxisome proliferator-activated receptor gamma coactivator 1-alpha
AICAR: 5-Aminoimidazole-4-carboxamide ribotide
AMPK: AMP-activated protein kinase
mPTP: Mitochondrial permeability transition pore
