# Supplementary figures and images for: Heat stress-induced mitochondrial damage and its impact on leukocyte function
Source: J Intensive Care. 2025 Nov 4;13:61. doi: 10.1186/s40560-025-00832-9 (PMC12584344; doi:10.1186/s40560-025-00832-9)

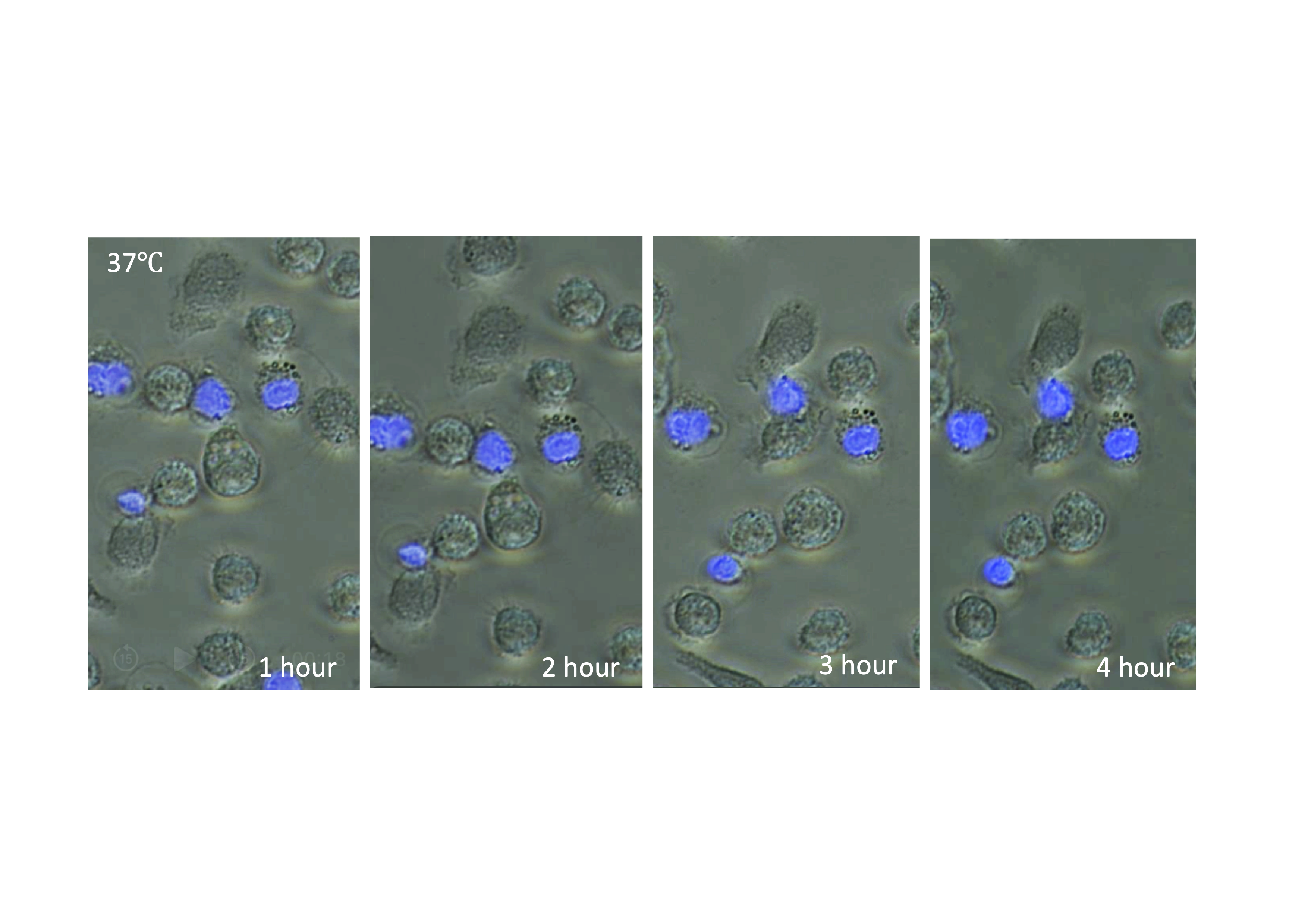

Supplement: Supplementary file 1 — Supplementary Material 1: Changes in leukocyte cell death. Time-lapse observation of leukocytes at 37℃ for 4 hours. Intracellular DNA was stained with DAPI (4‘,6-diamidino-2-phenylindole, blue), and phase-contrast imaging was used to assess cellular morphology. Both phase-contrast view and DAPI staining revealed minimal morphological changes for this period. [file 40560_2025_832_MOESM1_ESM.tiff]

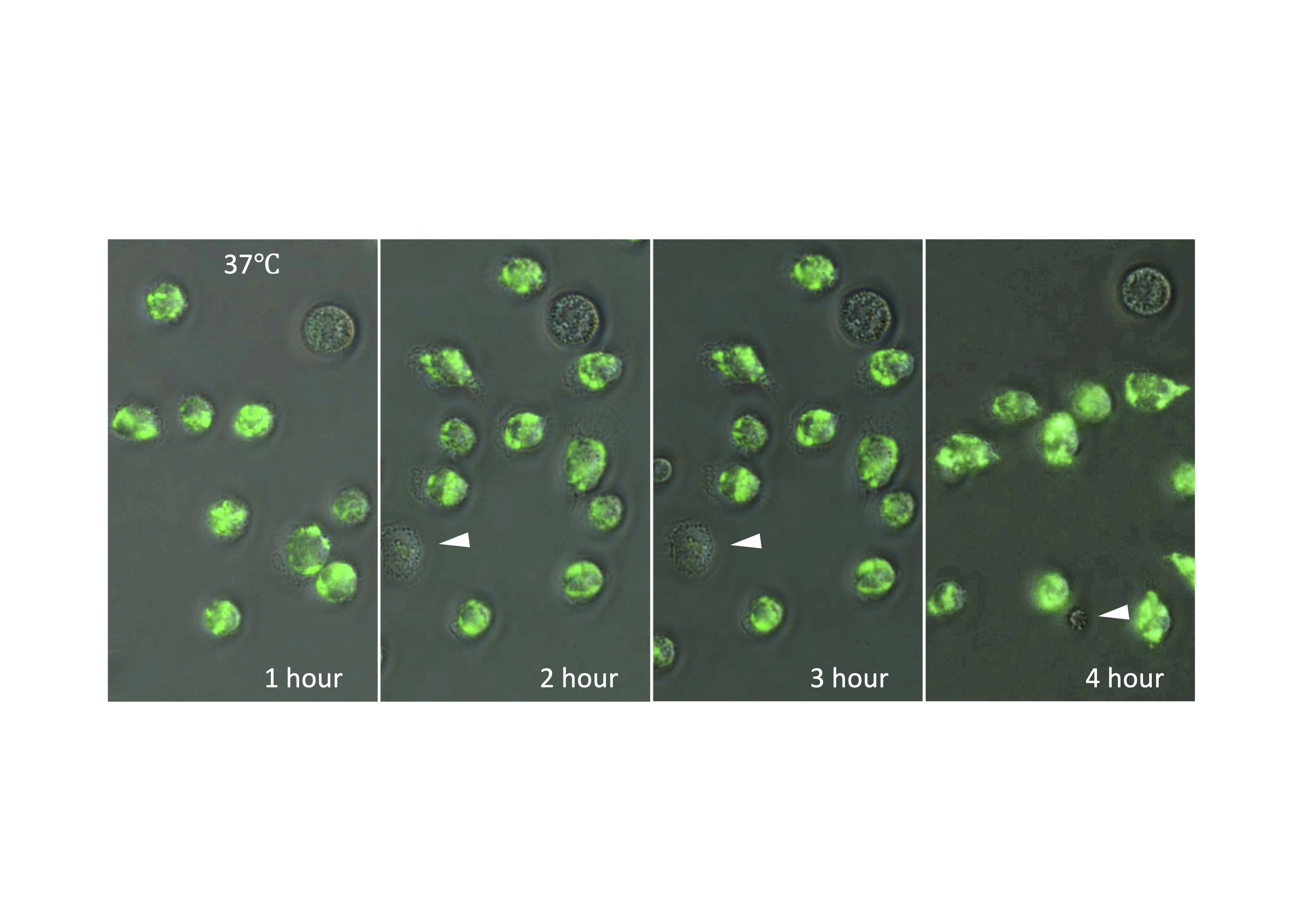

Supplement: Supplementary file 2 — Supplementary Material 2: Changes in mitochondrial staining in leukocytes. Mitochondria in leukocytes were visualized using MitoBright LT™ (Dojindo, Kumamoto, Japan) and observed for 4 hours at 37 ℃. This staining method allows for the assessment of mitochondrial distribution and function. As time went on, some cells showed a loss of mitochondrial staining (indicated by arrows). [file 40560_2025_832_MOESM2_ESM.tiff]

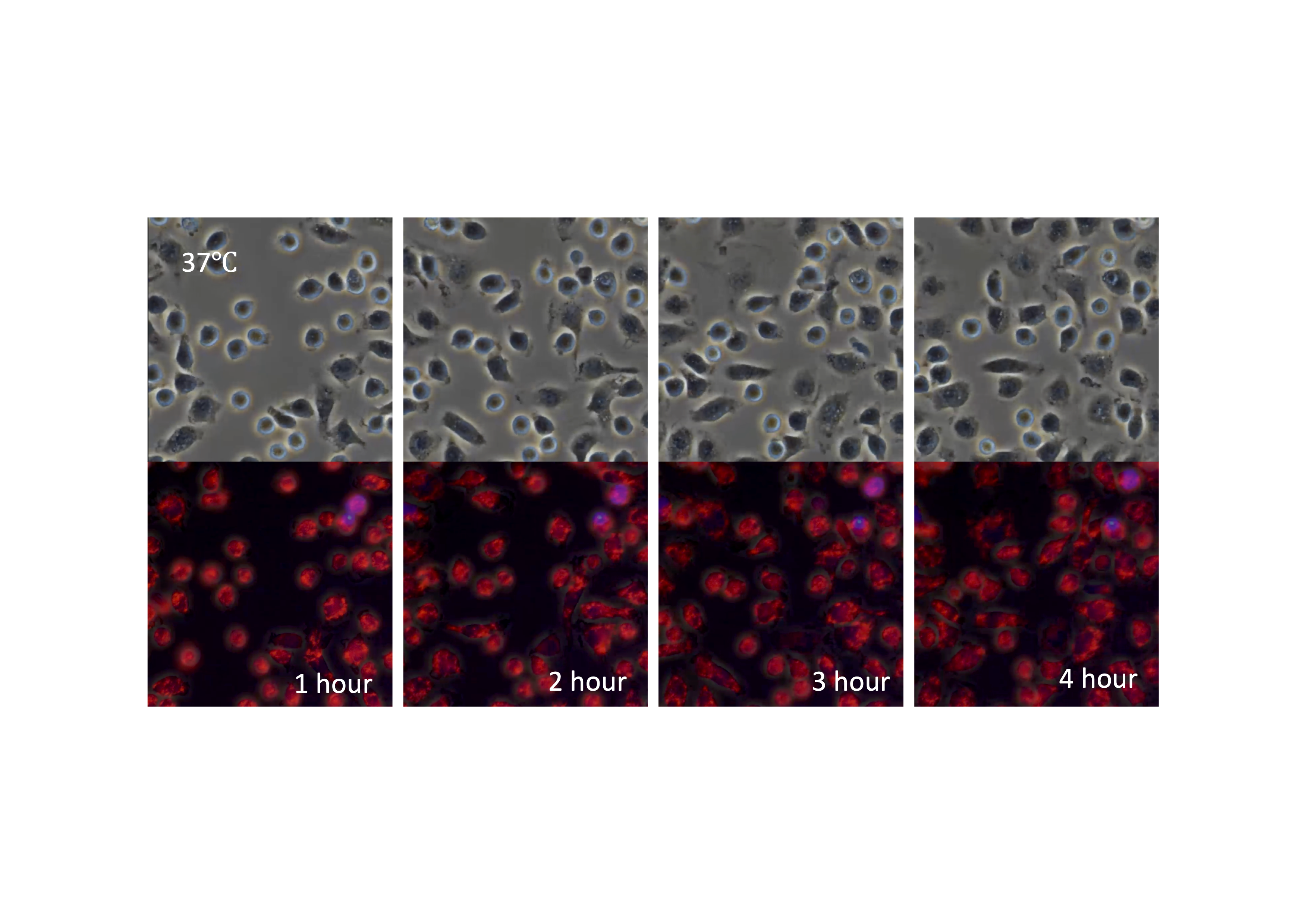

Supplement: Supplementary file 3 — Supplementary Material 3: Changes in mitochondrial membrane potential. Morphological changes of the leukocytes were observed under the microscope at 37℃ for 4 hours. No significant change was recognized (upper low). JC-1 (5,5',6,6'-tetrachloro-1,1',3,3'-tetraethylbenzimidazolylcarbocyanine iodide), a cationic fluorescent dye, was used to visualize membrane potential (Δψm). Under normal conditions, mitochondrial membrane potential was maintained for 4 hours (lower column). [file 40560_2025_832_MOESM3_ESM.tiff]

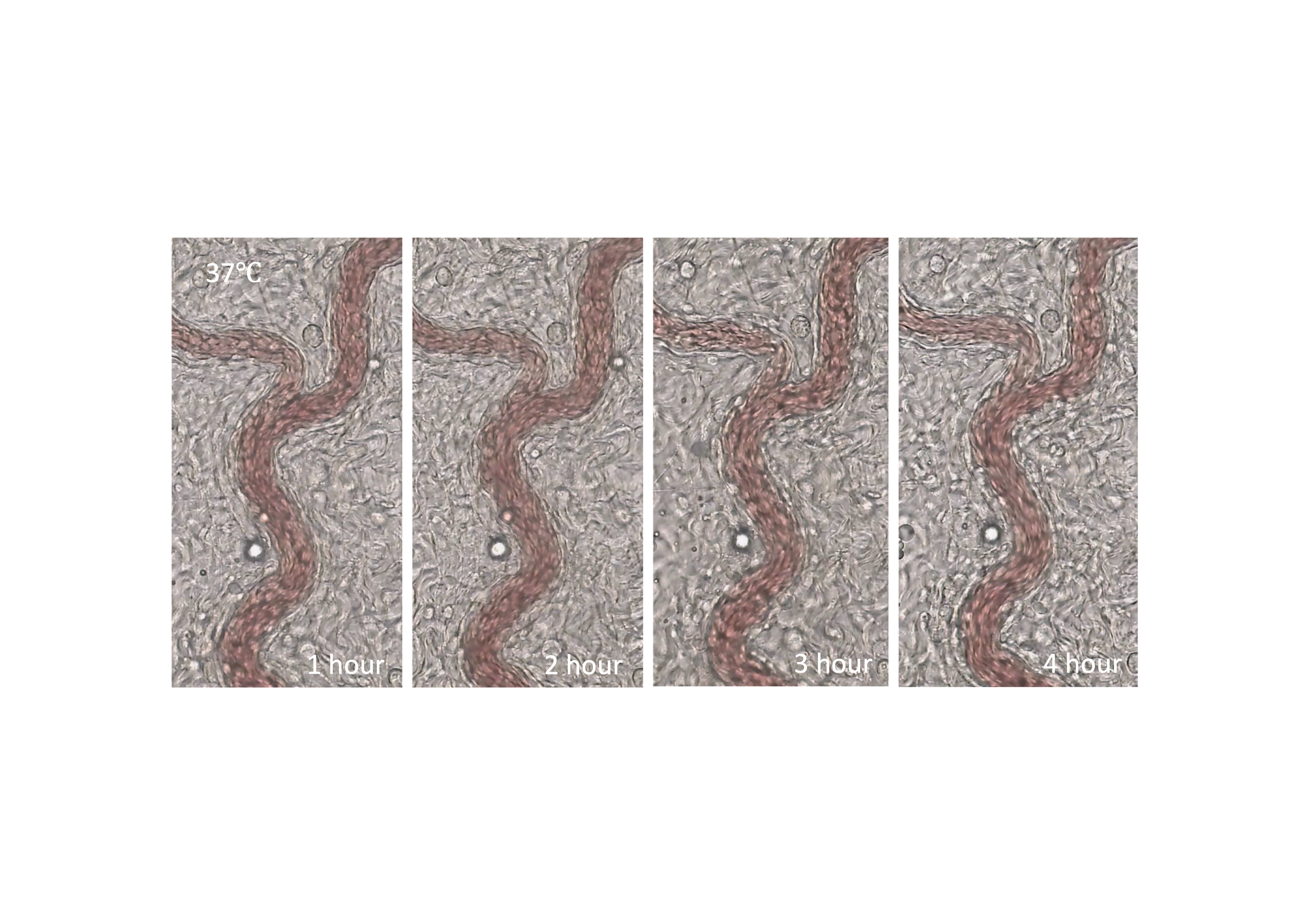

Supplement: Supplementary file 4 — Supplementary Material 4: Alterations in mesenteric microcirculation. Intravital microscopic observation revealed minimal changes in mesenteric flow in a rat. Blood flow appeared smooth with minimal leukocyte–endothelium interaction throughout the study period. [file 40560_2025_832_MOESM4_ESM.tiff]
